# A Retrospective Analysis of the Prognostic Factors and Adverse Events in the Treatment of Mucosal Melanoma in a Single Centre

**DOI:** 10.3390/jcm13164741

**Published:** 2024-08-13

**Authors:** Lambert Wesener, Victoria Hagelstein, Patrick Terheyden, Ewan A. Langan

**Affiliations:** 1 Clinic of Dermatology, Allergology and Venerology, University of Lübeck, 23562 Lübeck, Germany; lambert@wesener-hamburg.de (L.W.); victoria.hagelstein@uksh.de (V.H.); patrick.terheyden@uksh.de (P.T.); 2 Department of Dermatological Sciences, University of Manchester, Oxford Rd., Manchester M13 9PL, UK

**Keywords:** melanoma, mucosal melanoma, immune-related adverse events, adverse events, immune checkpoint inhibition, targeted therapy

## Abstract

**Background:** Despite the dramatic advances in the management of metastatic cutaneous melanoma, there remains no consensus-based, evidence-based strategy for the management of mucosal melanoma. The rare nature of the disease, its late clinical presentation, and distinct tumour biology all complicate efforts to optimise patient outcomes. **Methods:** To this end, we carried out a monocentric, retrospective analysis of all patients diagnosed with mucosal melanoma and treated between 2013 and 2021. Both tumour- and patient-specific characteristics were recorded, in addition to immune-related adverse events, in order to provide real-world data on disease progression, treatment efficacy, and the identification of prognostic markers. **Results:** A total of 20 patients were identified (14 females and 6 males), with a mean age at diagnosis of 65.9 years. The median follow-up was 3.9 years (95% CI 1.4–6.4 years) from the initiation of systemic therapy. The median OS in the entire cohort was 1.9 years (95% CI 0.5–3.3 years). Performance status, sex, body mass index, and the presence of brain metastases were not associated with poorer outcomes. However, serum lactate dehydrogenase levels (LDH) (*p* = 0.04) and an NRAS mutation were markers of a poor prognosis (*p* = 0.004). **Conclusuion:** There is a pressing need for real-world, prospective, and clinical trial data to inform the optimal management of mucosal melanoma, and data supporting the use of adjuvant and neo-adjuvant immunotherapy are currently lacking. However, an elevated LDH is a reliable, independent negative prognostic marker. Inter-disciplinary management remains essential in order to develop optimal treatment strategies.

## 1. Introduction

Mucosal melanoma is a rare, aggressive tumour that can arise at any mucosal surface and accounts for approximately 1% of all malignant melanomas [1]. The tumours arise from melanocytes and are distinct from cutaneous melanoma in terms of incidence, aetiology, and pathophysiology, the latter of which remains poorly understood [2,3] due to their rarity [4].

In contrast to cutaneous melanoma, exposure to ultraviolet radiation is not a significant risk factor for mucosal melanoma. Indeed, mucosal melanomas are classified according to the WHO classification of melanoma as tumours not consistently associated with cumulative solar damage [3,5]. The tumours less frequently harbour the BRAF V600 oncogene mutation and frequently present clinically at a later stage, which contributes to their poorer overall prognosis [6]. Early detection is further hampered by their anatomical location, which often results in a late presentation when the tumour has become symptomatic. Presenting symptoms include pressure effects, bleeding, pain, or discomfort. In fact, not only is the clinical presentation of mucosal melanomas delayed, but their surgical excision is frequently complicated by loco-regional spread at the time of diagnosis. Moreover, there is currently no consensus to inform optimal systemic therapy in the case of extensive loco-regional and/or distant metastases.

The current treatment landscape for metastatic mucosal melanoma is much less favourable than that for the treatment of cutaneous melanoma. The advent of immune checkpoint inhibition has revolutionised the treatment of cutaneous metastatic melanoma, with combined anti-programmed cell death protein 1 (anti-PD1) and anti-cytotoxic T-lymphocyte associated protein 4 (CTLA-4) inhibitors resulting in 5-year survival rates of 50%, with 75% of survivors treatment-free [7,8]. Indeed, the survival rates were stable with no new safety signals after 6 years [8]. At least part of the impressive response to immune checkpoint inhibitor therapy is due to the high tumour mutational burden seen in cutaneous melanoma [9].

Unfortunately, whole genome sequencing has revealed that mucosal melanoma has a markedly lower tumour mutational burden than cutaneous melanoma, presumably due to the lack of ultraviolet radiation as an aetiological factor, which explains the comparatively disappointing results of immune checkpoint inhibition [10,11,12]. The lack of prospective clinical trial data for mucosal melanoma has meant that the American Society for Clinical Oncology does not feel that even an expert opinion-based treatment recommendation is appropriate [13].

Of course, while treatment efficacy is the most important measure of response to immune checkpoint inhibitor therapy, the incidence of persistent and potentially life-threatening immune-related (irAE) events and other adverse events must also be taken into account. It is worth noting that combined nivolumab and ipilimumab treatment results in up to 60% of patients developing grade 3 and 4 (severe to life-threatening) adverse events [7]. A number of irAEs, particularly cutaneous irAEs, are associated with treatment response. For example, the development of vitiligo-like leukoderma has been observed in approximately 15% of patients treated with immune checkpoint inhibitors for metastatic cutaneous melanoma and in 9.3% of patients with mucosal melanoma. The development of vitiligo-like leukoderma was associated with a more favourable overall response in general and an improved overall response in particular [14]. Other studies have reported vitiligo in up to a quarter of anti-PD1 antibody-treated melanoma patients and a correlation with improved treatment outcomes [15].

Lactate dehydrogenase (LDH) is an established marker of poor prognosis in melanoma and is routinely measured in patients with metastatic disease [16,17]. Moreover, several meta-analyses examining the relationship between LDH and treatment response in cutaneous and uveal melanoma have been published since 2019 [18,19,20,21,22]. Elevated LDH was often associated with poorer overall survival, although these patients benefit significantly from immune checkpoint therapy and targeted therapy in those with a BRAF V600 mutation. The data for LDH as a prognostic marker in mucosal melanoma are less comprehensive, but a recent retrospective cohort study from 25 cancer centres in Australia, Europe, the USA, and Asia found elevated LDH to be associated with short progression-free and overall survival [23]. Almost one-third of the patients were Asian, and melanoma of the naso-oral mucosa was the most common site.

The development of a range of irAEs is associated with a favourable treatment response to immune checkpoint inhibition [24,25], which may at least be partially cancer-type- and treatment-specific [26]. Whether the treatment can safely be recommenced after the resolution of the irAEs, when treatment should be permanently discontinued, and when it is reasonable and safe to end treatment following disease remission remain open questions [27,28]. Although the tolerability of immune checkpoint inhibitors following the development of irAEs is acceptable overall, data supporting a superior clinical outcome are often retrospective [29]. Furthermore, given the low incidence of mucosal melanoma, data describing clinical outcomes after the development of irAEs or the tolerability of checkpoint inhibitors following treatment resumption are even more sparse [30]. There is an urgent need for robust real-world clinical data that can be used in meta-analyses to inform clinical decision-making.

Therefore, we carried out a retrospective analysis of patients with mucosal melanoma treated at a single university tertiary referral centre. The aim was to characterise the patient cohort in terms of treatment response, incidence, and type, or irAE, and to determine which factors may be associated with favourable treatment responses. In addition, we sought to identify if specific multi-modal treatment strategies improved clinical outcomes.

## 2. Methods

The data of all patients with primary mucosal melanoma who underwent systemic therapy between December 2013 and October 2021 were analysed in a retrospective monocentric study. This study received ethical approval from the University of Luebeck’s ethics committee (Reference number 20-387) and was performed according to the Declaration of Helsinki principles. Disease recurrence or progression (loco-regional versus distant) was established radiologically according to RECIST criteria or histologically.

The following patient characteristics were recorded: sex (male/female), performance status (ECOG), age (years), body mass index (kg/m^2^), and comorbidities (Charlson Comorbidity Index). In addition, the following disease criteria were recorded: tumour localisation and time of diagnosis, tumour stage, mutation status (BRAF/NRAS/cKit), and the presence of brain metastases (present/absent). In order to detail the treatment pathway, we also note the following: treatment setting (adjuvant/palliative), therapy (surgery, radiotherapy, chemotherapy and/or immunotherapy, targeted therapy), the treatment dose and number of cycles, in addition to adverse events, which were classified according to the common terminology criteria for adverse events (CTCAE) version 5.

In order to identify pre-treatment markers of response, we recorded leukocyte and lymphocyte, eosinophil, and thrombocyte counts. Renal function, antinuclear antibody status, and established tumour markers, S100 and LDH, were also documented prior to treatment [31]. Progression-free survival and overall survival were calculated.

## 3. Statistical Analysis

Data were analysed using Chi-Squared tests (categorical variables) or Kruskal–Wallis or Mann–Whitney U tests for continuous variables, depending on the distribution of the data. Data are expressed as median, range, standard deviation, and 95% confidence intervals. *p* values of <0.05 were considered significant. Univariate analysis was used to determine the influence of variables on median overall survival (Log-Rank method). A multivariate analysis was performed using variables that significantly affected overall survival or were clinically relevant. Given the number of patients, a maximum of two variables were included in the multivariate analyses a priori. Proportional hazard models (Cox Regression) were employed.

All data were analysed using the Statistical Package für Social Sciences (SPSS) program (IBM Corp., Released 2020; IBM SPSS Statistics for Windows, Version 27.0; IBM Corp., Armonk, NY, USA) and R software (Version 4.05.; R Core Team (2020); R: A Language and Environment for Statistical Computing). R Foundation for Statistical Computing, Vienna, Austria. URL: https://www.R-project.org/ accessed on 6 June 2024). Graphs were generated using Biorender (https://www.biorender.com accessed on 6 June 2024), and statistical advice was provided by the University of Luebeck’s Institute for Bioinformatics and Statistics.

## 4. Results

**Figure 1:**
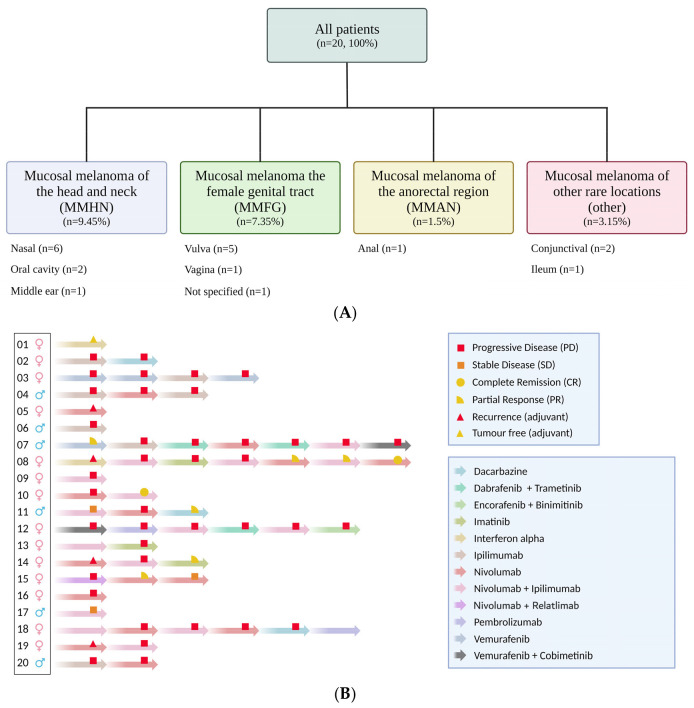

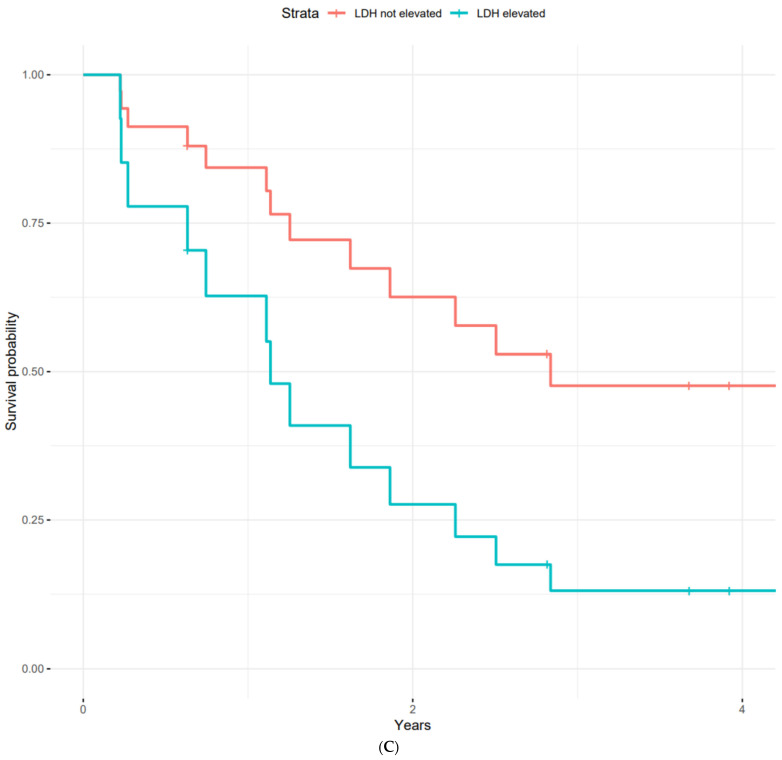
(**A**) Distribution of melanoma sites based on and modified from the classification of Heppt et al. [32]. (**B**) Lines of treatment and treatment responses. (**C**) The effect of serum LDH on overall survival.

There were a total of 20 patients (14 females and 6 males) with mucosal melanoma treated between 2013 and 2021. (Figure 1A) 19 of the patients were Caucasian; 1 patient was Asian. The mean body mass index was 23.7 (range 19.5–37.2), and the median Charlson comorbidity index was 3. Based on the classification of Heppt et al. [32], the majority of patients had mucosal melanoma of the head and neck, with melanoma of the anorectal region being the most infrequent.

The median age at first diagnosis was 65.9 years (range 53.2–93), with patients with mucosal melanoma of the female genital tract having the most advanced mean age at diagnosis (76.2 years, range 56–80.1 years). A total of 9 patients had Stage III disease, 10 had Stage IV, and in one case, the tumour stage was not adequately documented. A total of 5 patients had brain metastases or developed them during the course of their disease. Of those patients tested, 5 had a cKit mutation, 4 had a BRAF mutation, and 3 had an NRAS mutation. In terms of treatment setting, 70% were treated in the palliative- and 30% in the adjuvant treatment context. A total of 14 patients had died, and 6 were still alive at the end of the observation period. None of the patients were lost to follow-up.

Given the lack of an evidence-based consensus for the treatment of metastatic mucosal melanoma, it is not surprising that the majority of patients underwent a multi-modal treatment approach, including surgery, radiotherapy, targeted therapy, immunotherapy, and chemotherapy. The treatment lines and responses are detailed in Figure 1B. For example, 75% of patients underwent surgery prior to or following the initiation of systemic anti-tumour therapy. The same percentage of patients were treated with radiotherapy, while only 15% were treated with chemotherapy, and all patients underwent immunotherapy during their cancer treatment. This sequence of treatment therapies reflects the historical treatment landscape and the availability of anti-PD1 treatment in the adjuvant setting. The median interval between the first-line and second-line therapies was only 4.1 months (range 1–24.7 months).

Having recorded the patient, disease, and treatment characteristics, we next analysed the influence of several variables on overall survival (OS). The median follow-up was 3.9 years (95% CI 1.4–6.4 years) from the initiation of systemic therapy. The median OS in the entire cohort was only 1.9 years (95% CI 0.5–3.3 years). There was no significant effect of sex, performance status, comorbidities, BMI, or the presence of brain metastases (Table 1).

We also examined the effect of tumour-specific factors on OS. Specifically, we investigated the effect of mutation status and serum lactate dehydrogenase (LDH) concentrations (Table 2). An NRAS mutation was associated with significantly worse overall survival.

Finally, a Cox-Regression multivariate analysis was performed with the serum LDH and the presence of brain metastases as pre-selected factors based on the univariate analysis. An elevated LDH showed a hazard ratio of 3.45 (95% CI 1.08–10.7, *p* = 0.04) and 0.44 (95% CI 0.11–1.67, *p* = 0.23) for the presence of brain metastases. Therefore, elevated LDH was confirmed as an independent risk factor for mortality (Figure 1C).

Turning to the development of irAEs, three-quarters of the patients developed at least one adverse event, and 40% (n = 8) developed severe irAEs (CTCAE grade ≥ 3). Indeed, one patient died from irMyocarditis (CTCAE grade 5). The most frequent irAE was colitis (10 cases), although these were almost exclusively mild to moderate in severity (90% CTCAE grade 1–2), although recurrent in some patients. A detailed description of the adverse events is seen in Table 3 and Table 4.

## 5. Discussion

This retrospective monocentric analysis confirms the poor prognosis associated with metastatic mucosal melanoma, despite the advent of immune checkpoint immunotherapy. The diagnosis occurred most commonly in the seventh and eighth decades of life, and the median OS was under 2 years. Almost 50% of the patients had a mucosal melanoma affecting the head and neck area, with just over a third of patients having a mucosal melanoma of the female genital tract. The median time from first-line to second-line therapy was 4.1 months (range 1–24.7 months) and just 3.4 months from second-line to third-line therapy (range 1–12.4 months). This relatively rapid sequence between lines of systemic therapy reflects the aggressive nature of the disease and the difficulty in achieving disease control and remission.

Consistent with the literature and registry data, females with mucosal melanoma were over-represented [32,33]. Females made up 70% of our cohort, and this at least partially reflects the incidence of mucosal melanoma of the female genital tract [34]. According to Pandey et al. [34], approximately 50% of the patients present with a nodule or ulcer with or without pigmentation, but all of the patients with vaginal melanoma had bleeding, and three-quarters reported pain. Given the rapid progression of mucosal melanoma and the median 2-year disease-free survival rate of only 13.2%, rapid assessment of females with vaginal bleeding is key to preventing diagnostic delay and potentially improving overall survival.

Despite the relatively small cohort in our study, it is reassuring to note the similarities with the multi-centric analysis of Heppt et al. [32], which supports the representative nature of our cohort. Indeed, the average age at diagnosis reported by Heppt et al. [32] was 66 years, which compares well to the equivalent figure of 65.9 years in our cohort.

Unfortunately, Heppt et al. [32] did not examine the effect of body mass index on survival outcomes in patients with mucosal melanoma. Although this may initially seem surprising, the so-called obesity paradox in immunotherapy treatment was first widely reported a year later. In a retrospective multicohort analysis of over 2000 patients with metastatic melanoma, McQuade et al. [35] reported that obesity was associated with both improved progression-free and overall survival compared to that in patients with a normal BMI, an effect seen mainly in male patients treated with targeted or immune therapy.

However, it is worth noting that patients with mucosal melanoma were not specifically mentioned in the publication and were actively excluded from at least one of the cohorts, and patients with brain metastases were excluded from several of the cohorts [35]. There was no effect of BMI on overall survival in our cohort, although it should be borne in mind that over 40% of patients in the cohort had a BMI > 25 kg/m^2^.

In fact, the existence and clinical significance of the “obesity paradox” have recently been called into question in meta-analyses. For example, Donnelly et al. [36] found that any potential relationship between BMI and survival may not only be dependent on the immunotherapy regimen (combined immunotherapy versus monotherapy) but also on whether it was administered in the first-line or subsequent treatment-line setting. It is unclear whether patients with mucosal melanoma were included in the analyses. Nonetheless, given that the median time from first-line to second-line therapy in our cohort was only 4.1 months, it would be important to clarify whether any effect of BMI is treatment type- and setting-dependent in prospective studies. Moreover, there is a pressing need for specific data examining the effect of BMI on treatment response in the context of mucosal melanoma. Until these data are available, caution should be exercised before drawing conclusions that could negatively impact patient care [36,37,38].

In the prospective multivariate analysis, we could confirm that an elevated serum LDH concentration was an independent prognostic factor for a less favourable outcome. This supports the findings of Cui et al. [39], who also reported LDH to be an independent prognostic factor in a multi-factorial analysis of over 700 patients. At the same time, only approximately 6% of the patients in that study underwent treatment with immunotherapy, in contrast to of the higher proportion of patients in our study. Therefore, despite the advances in treatment, an elevated serum LDH concentration remains a robust negative prognostic marker. Indeed, several subsequent studies, including national registry-based studies, have confirmed the association [40,41], even in patients treated with immune checkpoint inhibitors [42].

More surprising in our study was the lack of a significant difference in OS depending on the presence of brain metastases. This may be partially due to the over-representation of patients with brain metastases and the fact that 80% of the patients (n = 4) without brain metastases progressed and should, therefore, be interpreted with caution. Indeed, mucosal melanoma itself has been identified as an adverse prognostic factor for Asian patients with brain metastases [43]. In general, the incidence of brain metastases in mucosal melanoma is less than that seen in cutaneous melanoma [44,45]. Nevertheless, the presence of brain metastases was a negative prognostic marker in oral melanoma [46]. Routine brain imaging is clearly indicated in the management and surveillance of metastatic mucosal melanoma. Prospective data are required to determine the extent to which brain metastases may negatively impact prognosis and whether prognostic factors apply to mucosal melanoma as a whole or are site-dependent [39].

This study provides a comprehensive description of the AEs and irAEs during and following treatment for mucosal melanoma (Table 4). As seen in Figure 1B, the patients had multiple lines of treatment, often multi-modal in nature, switching between immunotherapy, targeted therapy, and even chemotherapy. Furthermore, irAEs can occur many months, even years, after the cessation of treatment. The size of the cohort, the multiple lines of treatment, and the potential latency of irAEs prevented an analysis of whether age- and/or irAEs influenced progressive free- or overall survival in our cohort. However, these are important questions that should be addressed in clinical trials and prospective studies.

This study’s strengths include the generation of real-world data, which captures the multi-modal, multi-disciplinary approach to the management of mucosal melanoma. Moreover, the cohort includes patients treated with immune checkpoint therapy, which confirms the independent prognostic value of measuring serum LDH. Its conclusions are limited by its retrospective and monocentric nature. Moreover, given the rarity of mucosal melanoma and the size of the cohort, caution should be exercised before extrapolating our results to patients with mucosal melanoma in general. Furthermore, the treatments selected were based on those available at the time and would certainly be different today. For example, the use of immune checkpoint inhibitors has largely replaced the use of interferon-alpha in the adjuvant setting. Nevertheless, the data may be useful for evaluating real-world data on newer treatments by providing a historical comparator.

An evidence-based, guideline-supported management strategy for mucosal melanoma remains elusive. Moreover, data supporting the routine adjuvant use of anti-PD1 immune checkpoint inhibitors are scant, and their efficacy is not comparable to that in the treatment of cutaneous melanoma [47]. There is a pressing need for innovative treatment options, and neo-adjuvant therapy may hold some promise [48,49]. Given the rare nature of the disease, real-world and national registry data, supported by prospective clinical trials, are all essential to identifying new prognostic markers and treatment strategies.

## Figures and Tables

**Table 1:** The effect of patient-dependent factors on OS.

Variable	Median OS (Years)	95% Confidence Interval	Significant? *p* < 0.05
Sex			
Male	2.3	0.7–3.8	
Female	1.3	0–2.5	no
ECOG Status			
0	1.9	0.8–3.0	
≥1	4.3	0–10.5	no
Comorbidities			
CCI < 3	1.6	0.2–3.9	
CCI ≥ 3	1.4	0–4.6	no
Body mass index			
≤25	2.3	0–5.2	
>25	1.6	0.6–2.7	no
CNS metastases			
Yes	2.5	0–5.2	
No	1.9	0–3.2	no

**Table 2:** The effect of disease-dependent factors on OS.

Variable	Median OS (Years)	95% Confidence Interval	Significant? *p* < 0.05
BRAF mutation			
Positive	2.3	1.0–3.5	
Wild type	1.6	0.7–2.5	no
NRAS mutation			
Positive	0.2	0.22–0.24	
Wild type	1.9	0.7–3.0	yes (*p* = 0.004)
cKit mutation			
Positive	4.2	N/A	
Wild type	1.6	0.8–2.4	no
LDH			
Normal	2.5	0.4–4.6	
Elevated	1.1	0–2.2	no

**Table 3:** Detailed description of the adverse events recorded in the cohort.

	Cobimetinib, Vermurafenib	Dabrafenib, Trametinib	Dacarbazine	Imatinib	Ipilimumab	Ipilimumab, Nivolumab	Nivolumab	Total
Alanine aminotransferase increased (hepatitis)	0	0	0	0	1	3	0	4
Alkaline phosphatase increased	1	0	0	0	0	0	0	1
Alopecia	0	0	0	1	0	0	0	1
Anaemia	0	0	0	1	0	0	0	1
Arthritis	0	0	0	0	0	1	0	1
CPK increased	1	0	0	0	0	0	1	2
Creatinine increased (nephritis)	1	0	0	0	0	1	0	2
Diarrhoea (colitis)	0	0	0	0	2	6	2	10
Facial nerve disorder	0	0	0	0	0	1	0	1
Fever	0	1	0	0	0	0	0	1
Hyperglycemia	0	0	0	0	0	1	0	1
Hyperparathyroidism	0	0	0	0	0	0	1	1
Hyperthyroidism	0	0	0	0	0	2	0	2
Hypophysitis	0	0	0	0	0	1	1	2
Lipase increased	2	0	0	0	0	1	0	3
Myocarditis	0	0	0	0	0	1	0	1
Nausea	0	0	0	0	0	1	0	1
Peripheral motor neuropathy	0	0	1	0	0	0	0	1
Platelet count decreased	0	0	1	0	0	0	0	1
Pneumonitis	1	0	0	0	0	0	1	2
Rash maculo-papular	0	0	0	0	1	2	0	3
Serum amylase increased	1	0	0	0	0	0	0	1
Skin hypopigmentation (Vitiligo)	0	0	0	0	0	2	0	2
Uveitis	0	0	0	0	0	0	1	1
White blood cells decreased	0	0	0	1	0	0	0	1
Gesamt	7	1	2	3	4	23	7	47

**Table 4:** A comprehensive list of irAEs following administration of immunotherapy. There were no grade 5 irAEs following immunotherapy when it was administered for the first time.

Immune-Related Adverse Event (irAE)	None	Grade 1–2	Grade 3–4	Grade 5	Total
Alanine aminotransferase elevated (hepatitis)	17	0	3	0	3
Alkaline phosphatase elevated	19	1	0	0	1
Alopecia	20	0	0	0	0
Anaemia	20	0	0	0	0
Arthritis	20	0	0	0	0
Creatine phosphokinase elevated	19	1	0	0	1
Creatinine elevated (nephritis)	18	2	0	0	2
Diarrhoea	15	4	1	0	5
Facial nerve paralysis	19	1	0	0	1
Fever	20	0	0	0	0
Hyperglycaemia	20	0	0	0	0
Hyperparathyroidism	20	0	0	0	0
Hyperthyroidism	19	0	0	0	1
Hypophysitis	20	0	0	0	0
Lipase elevated	18	0	2	0	2
Myocarditis	20	0	0	0	0
Nausea	20	0	0	0	0
Peripheral motor neuropathy	20	0	0	0	0
Platelet count decreased	20	0	0	0	0
Pneumonitis	20	0	0	0	0
Maculo-papular rash	19	1	0	0	1
Serum amylase elevated	19	1	0	0	1
Skin hypopigmentation (vitiligo)	20	0	0	0	0
Uveitis	20	0	0	0	0
White blood cell count decreased	20	0	0	0	0

## Data Availability

The data presented in this study are available on request from the corresponding author.
